# Continuous OFT-Based Behavioral Phenotyping Reveals Neural Stem Cell-Associated Shifts in Depression-like Mice

**DOI:** 10.3390/brainsci16090909

**Published:** 2026-08-26

**Authors:** Han Wang, Zhaoming Liu, Xianjie Li, Lin Fu, Tingxin Lu, Dantong Wang, Guanzhen Lin, Caixia Wu

**Affiliations:** 1 Medical Experiment and Health Evaluation Center, Institute of Bio and Medical Engineering, Guangdong Academy of Sciences, Guangzhou 510316, China; wanghan0603@outlook.com (H.W.); lixianjiejx@163.com (X.L.); lindexiaoqie@163.com (L.F.); 18320093708@163.com (T.L.); glinae@connect.ust.hk (G.L.); 2 National Engineering Research Center for Healthcare Devices, Guangzhou 510316, China; 3 Guangzhou Institutes of Biomedicine and Health, Chinese Academy of Sciences, Guangzhou 510530, China; liu_zhaoming@gibh.ac.cn; 4 School of Microelectronics, South China University of Technology, Guangzhou 510641, China; 18811635669@163.com; 5 Institute of Biological and Medical Engineering, Guangdong Academy of Sciences, Guangzhou 510316, China

**Keywords:** depression-like behavior, behavioral phenotyping, open field test, machine learning, neuroinflammation, neural stem cells

## Abstract

**Background:** Depression-like phenotypes in animal models often show graded behavioral variation rather than uniform group-wise changes, yet treatment effects are still commonly evaluated using categorical comparisons. In particular, it remains difficult to determine whether treated animals retain model-like behavioral features or shift toward a more control-like state. Here, we developed a continuous behavioral phenotyping framework based on multidimensional open-field-test (OFT) features to quantify model-like behavioral states and treatment-associated behavioral shifts. **Methods:** In the present dataset, untreated CUMS- and LPS-induced mice jointly defined a shared model-like behavioral state characterized by reduced locomotor activity and altered spatial exploration. By integrating principal component analysis with k-nearest-neighbor-based behavioral inference, we derived two complementary indices: Δd, representing the relative distance of an animal from the model-like versus control-like behavioral centroids, and p_depression, a kNN-derived score representing similarity to the untreated model-like reference state. These indices were used to position individual animals along a spectrum between control-like and model-like behavioral states. **Results:** Higher p_depression and lower Δd indicated OFT phenotypes more similar to untreated model mice. Using this framework, NSC-treated animals showed treatment-associated shifts away from model-like behavioral patterns and toward more control-like states, while preserving substantial inter-individual variability. Behavioral scores also showed group-level correspondence with neuroinflammatory markers, suggesting preliminary biological relevance. Because the primary model-specific biological comparisons were based on a limited subgroup size (*n* = 4 per group), the observed NSC-associated treatment effects should be regarded as exploratory and require confirmation in larger, prospectively powered cohorts. **Conclusions:** Together, these findings support continuous OFT-based behavioral phenotyping as a promising exploratory approach for evaluating treatment-associated behavioral change and phenotypic heterogeneity in preclinical depression research.

## 1. Introduction

Depression is a heterogeneous neuropsychiatric disorder, and depression-like behaviors in animal models often vary along a spectrum rather than as uniform group-wise changes [1,2,3]. In preclinical studies, behavioral assays such as the OFT, forced swim test (FST), and tail suspension test (TST) are widely used to evaluate depression-like phenotypes [4,5,6,7,8]. Treatment effects are still most often interpreted using categorical comparisons, such as control versus model or model versus treatment, which may obscure intermediate behavioral states, partial recovery, and inter-individual variability [9,10]. As a result, it is often difficult to determine whether treated animals truly move away from a model-like behavioral state or simply show modest group-level improvement.

Among commonly used paradigms, LPS-induced mice provide a robust inflammation-associated depression-like phenotype whereas CUMS remains a widely used paradigm for modeling chronic stress-related depression-like behavior [11,12,13]. Although CUMS and LPS models differ in their primary biological mechanisms, with CUMS representing chronic stress-related adaptation and LPS representing inflammation-associated behavioral alterations, both paradigms can induce partially overlapping OFT phenotypes. Therefore, these models provide complementary platforms to examine whether multidimensional behavioral features capture a shared model-like behavioral state across distinct induction procedures [14,15]. For treatment-oriented studies, what is often most relevant is not merely whether group means differ, but whether the behavioral profile of an individual animal remains closer to an untreated model-like state or shifts toward a more control-like exploratory pattern after intervention [16,17].

Recent data-driven approaches offer new opportunities to address this problem [18]. Dimensionality reduction methods such as PCA can uncover latent structure within multidimensional behavioral datasets, whereas machine-learning-based approaches can estimate similarity to predefined behavioral states [19,20]. In particular, k-nearest-neighbor-based behavioral inference is well suited for classifying whether a given behavioral profile more closely resembles control-like or model-like reference patterns. When combined with interpretable feature extraction, such approaches can convert multidimensional OFT readouts into quantitative behavioral coordinates while preserving inter-individual variation.

In parallel, neuroinflammation has been increasingly implicated in the pathophysiology of depression [21,22,23]. Neuroinflammatory activation and oxidative imbalance have also been implicated in stress- and inflammation-associated depression-like behavioral alterations, providing a biological rationale for examining inflammatory and oxidative-stress-related markers alongside behavioral phenotypes. Pro-inflammatory cytokines such as interleukin-1β (IL-1β) and tumor necrosis factor-α (TNF-α), together with anti-inflammatory mediators including IL-10, have been linked to altered behavioral outcomes in both clinical and preclinical studies [24,25]. Accordingly, IL-1β, TNF-α, and IL-10 were selected to characterize pro- and anti-inflammatory regulation, whereas GSH-Px and T-SOD were used as indicators of antioxidant defense and MDA as an index of lipid peroxidation and oxidative damage. These markers were included to determine whether behavioral alterations occurred in parallel with broader inflammatory and oxidative-stress-related changes. Whether graded behavioral variation corresponds to inflammatory-state differences remains insufficiently characterized [26], particularly when behavior is represented as a continuous rather than categorical phenotype [27,28]. Clarifying this correspondence would improve the biological interpretability of quantitative behavioral phenotyping [29,30].

Neural stem cells (NSCs) represent an emerging biologically distinct intervention strategy with potential relevance to neurological and neuropsychiatric disorders. NSCs are self-renewing, multipotent neural progenitor cells capable of generating neural lineage cells and releasing trophic factors that support neural microenvironment regulation [31,32]. Beyond their regenerative potential, increasing evidence suggests that NSC-based interventions may exert immunomodulatory, neuroprotective, and plasticity-promoting effects through regulation of inflammatory responses, oxidative stress, and neural repair processes [32]. Previous preclinical studies using NSCs or NSC-derived products have demonstrated beneficial effects in models of neurological injury, neurodegeneration, and inflammation-associated brain dysfunction, suggesting potential translational relevance [33]. However, unlike conventional antidepressant drugs that primarily target neurotransmitter systems, NSC-based approaches represent a regenerative and immunomodulatory strategy, and their effects on multidimensional behavioral phenotypes remain insufficiently characterized [34].

In this study, we developed a continuous OFT-based behavioral phenotyping framework to quantify exploratory and anxiety-related behavioral variation and characterize NSC-associated behavioral shifts in mice. Using control animals and untreated depression-model animals, including CUMS- and LPS-induced mice, as reference states, we integrated PCA with k-nearest-neighbor-based inference to derive two complementary indices, Δd and p_depression (a model-like behavioral probability derived from reference-state similarity). We then used this framework to determine whether NSC-treated animals shifted away from untreated model-like behavioral patterns and toward more control-like exploratory states, and whether these behavioral scores corresponded to neuroinflammatory profiles. We hypothesized that this approach would provide a more sensitive and interpretable evaluation of treatment-associated behavioral changes than conventional categorical comparisons.

## 2. Materials and Methods

### 2.1. Animals and Experimental Procedures

#### 2.1.1. Animals and Experimental Design

A total of 69 male C57BL/6 mice (8 weeks old, 18–22 g) were included in this study (Table 1). Fifty-six mice were obtained as the primary experimental cohort, and an additional 13 untreated mice from an independent batch were included as normal-reference animals.

Animals were housed under controlled conditions (25 °C, 50% relative humidity, and a 12 h light/12 h dark cycle) with free access to food and water. The light/dark cycle was maintained to simulate a regular circadian environment. All behavioral tests were conducted during the daytime light phase between 9:00 and 17:00, and the same testing window was maintained across experimental groups to minimize circadian-related variation. After a 7-day acclimation period, animals were randomly assigned to experimental groups.

**Figure 1:**
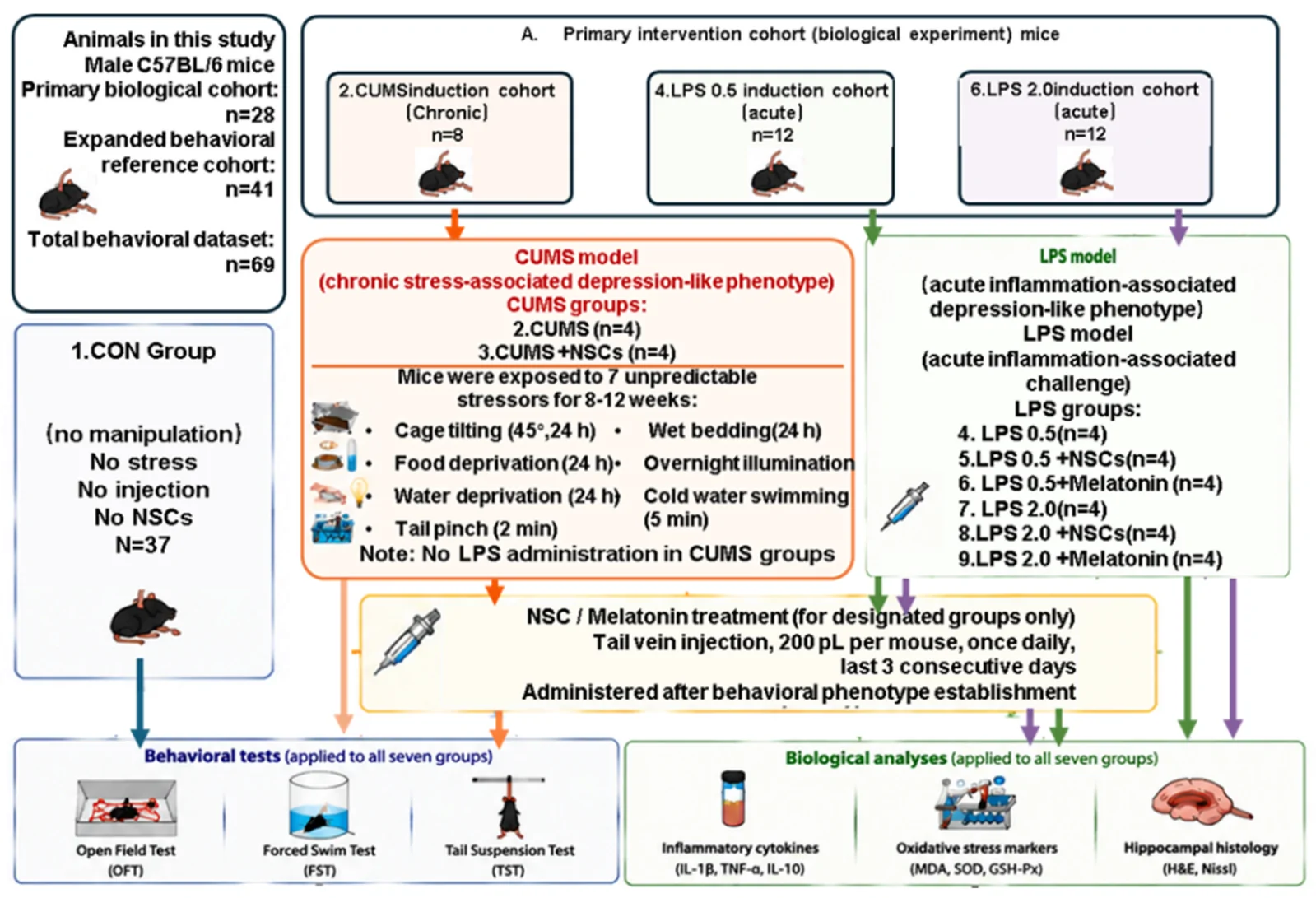
Experimental design for OFT-based evaluation of NSC-treated depression-like mice using untreated CUMS- and LPS-induced animals as model-like reference groups. Male C57BL/6 mice were assigned to seven groups: control (CON), CUMS, CUMS+NSCs, LPS0.5, LPS0.5+NSCs, LPS2.0, and LPS2.0+NSCs. Depression-like states were induced using chronic unpredictable mild stress (CUMS) or LPS administration. In the present study, untreated CUMS- and LPS-induced mice jointly defined the model-like reference class for OFT-based scoring, whereas NSC-treated groups were used to evaluate treatment-associated behavioral shifts. NSCs were delivered by tail vein injection after model establishment. Behavioral phenotyping included the OFT, FST, and TST. Hippocampal tissues were subsequently collected for histopathological and biochemical analyses, including inflammatory and oxidative-stress-related markers. OFT-derived features were further used for dimensionality reduction, continuous behavioral scoring, and treatment-response assessment. The expanded behavioral cohort additionally included melatonin(10 mM in DMSO; Aladdin Scientific, Shanghai, China; Cat. No. M408915)-treated LPS animals as a reference intervention group. These animals were analyzed only in the expanded cohort and were not included in the primary seven-group NSC-specific comparisons.

The primary experimental cohort consisted of normal-reference animals, untreated depression-model animals, and intervention groups. For the primary behavioral comparisons, seven predefined experimental groups were analyzed with *n* = 4 animals per group (Figure 1). Biochemical measurements were performed on the available hippocampal tissue samples, and the exact number of biological samples included in each biochemical analysis is reported in the corresponding figure legend:

Control (CON), LPS 0.5 mg/kg (LPS0.5), LPS 0.5 mg/kg + neural stem cells (LPS0.5+NSCs), LPS 2.0 mg/kg (LPS2.0), LPS 2.0 mg/kg + NSCs (LPS2.0+NSCs), CUMS, and CUMS + NSCs (CUMS+NSCs).

No formal a priori power calculation was performed when the original exploratory experiment was designed. The subgroup size of *n* = 4 was therefore not selected to achieve a predefined statistical power and the corresponding biological comparisons were treated as exploratory. Future confirmatory studies will require prospective sample-size estimation based on predefined primary outcomes and expected effect sizes.

No animals were excluded after allocation, and no data points were removed based on behavioral outcomes or statistical considerations. Animal allocation, inclusion/exclusion criteria, and analysis populations were reported in accordance with the ARRIVE 2.0 guidelines [35].

All experimental procedures were approved by the Institutional Animal Care and Use Committee of the Institute of Biomedical and Health Engineering, Guangdong Academy of Sciences (Ethics Approval No. K202402-162-182; Approval Date: 24 August 2024). Behavioral testing and sample collection were performed according to the approved experimental protocol.

For the continuous behavioral phenotyping analysis, all available OFT datasets were integrated into three analysis populations:

The broader behavioral cohort included melatonin-treated animals as a reference intervention. Melatonin was selected because previous studies have demonstrated antidepressant-like effects in experimental depression models [36,37]. Animals received melatonin at 10 mg/kg via tail vein injection using the same administration route as NSC treatment. These animals were included only in the secondary expanded-cohort robustness analysis and were not involved in the primary seven-group NSC-specific biological comparisons.

#### 2.1.2. Establishment of Depression-like Models

Two established mouse paradigms were used to induce depression-like behavioral alterations: CUMS and LPS-induced inflammation-associated behavioral changes.

##### CUMS Model

The CUMS model was established according to previously described procedures with minor modifications [2]. Mice were exposed to seven stressors in an unpredictable sequence, including cage tilting, food deprivation, water deprivation, tail clipping, wet bedding, continuous illumination for 24 h, and forced swimming in cold water. Stressors were randomly scheduled and repeated throughout the modeling period to minimize adaptation. Additionally, 24 h continuous illumination was applied as an unpredictable stressor during CUMS exposure and was not part of the standard housing condition.

##### LPS-Induced Model

For LPS-induced behavioral alterations, lipopolysaccharide (LPS; Sigma-Aldrich, St. Louis, MO, USA)was freshly prepared in sterile normal saline and administered intraperitoneally at doses of 0.5 mg/kg or 2.0 mg/kg. The administration schedule was designed according to the corresponding experimental groups. Behavioral assessments were subsequently performed following LPS exposure [38].

#### 2.1.3. NSC Preparation, Quality Control, and Administration

NSCs were obtained from the Guangzhou Institutes of Biomedicine and Health, Chinese Academy of Sciences. Cells were cultured under standard conditions and collected at passages 3–5. Cell viability was confirmed to be greater than 80% before administration.

NSCs were suspended at a concentration of 2 × 10^7^ cells/mL, and each mouse received 4 × 10^6^ cells via tail vein injection once daily for three consecutive days. Corresponding control groups received an equal volume of phosphate-buffered saline (PBS; Meilunbio, Dalian, China; Cat. No. MA0015).

### 2.2. Behavioral and Biochemical Assays

#### 2.2.1. Open Field Test (OFT)

The OFT was performed to evaluate locomotor activity and exploratory behavior [39]. Behavioral trajectories were recorded using EthoVision XT 17 (Noldus Information Technology, Wageningen, The Netherlands). Each mouse was placed individually in an open-field arena (40 × 40 cm) and allowed to explore freely for 6 min.

The arena was divided into a 3 × 3 grid consisting of nine zones (zones 1–9), with zone 5 defined as the central region. Behavioral parameters, including total distance traveled, movement time, mean velocity, zone-specific time ratio, and distance ratio, were extracted from the recorded trajectories for subsequent analysis.

#### 2.2.2. Tail Suspension Test (TST)

The TST was performed to evaluate stress-related behavioral responses [40]. Each mouse was suspended individually by the tail using adhesive tape, with the body positioned approximately 50 cm above the floor. The total testing duration was 6 min, and immobility time was recorded during the final 4 min.

#### 2.2.3. Forced Swimming Test (FST)

The FST was conducted using a transparent cylindrical container filled with water maintained at an appropriate temperature [28]. Mice were placed individually in the container for a 6-min session, and immobility duration during the final 4 min was quantified. FST and TST were used as complementary behavioral assessments to support model characterization rather than as direct components of the continuous OFT-based prediction framework.

#### 2.2.4. Histological Analysis

Following behavioral testing, brain tissues were collected and processed for hematoxylin and eosin (H&E) staining. Representative sections from the hippocampal CA1 region and dentate gyrus (DG) were examined qualitatively for general morphological features and cellular arrangement. No formal blinded morphometric scoring or quantitative histopathological analysis was performed. Therefore, these observations were used only as descriptive supportive evidence and were not subjected to inferential statistical analysis.

#### 2.2.5. Biochemical Measurements

Hippocampal tissues were collected for biochemical analysis. Hippocampal inflammatory and oxidative-stress-related markers were measured to evaluate biological changes associated with model induction and treatment response. Pro-inflammatory cytokines, including IL-1β and TNF-α, anti-inflammatory cytokine interleukin-10 (IL-10), antioxidant enzymes glutathione peroxidase (GSH-Px) and total superoxide dismutase (T-SOD), and lipid peroxidation marker malondialdehyde (MDA), were quantified.

Oxidative stress-related parameters, including GSH-Px, T-SOD, and MDA, were also quantified according to the experimental protocols.

Biochemical assays were conducted using the available hippocampal tissue samples from each experimental condition. Because not all animals were included in every biochemical assay, the exact biological sample size for each group is specified in the corresponding figure legend. When repeated technical measurements were available for the same biological sample, these measurements were averaged before statistical analysis so that each animal contributed a single value for each biochemical marker. Biological sample size therefore refers to the number of independent animals rather than the number of technical measurements.

### 2.3. Data Preprocessing and Feature Extraction

Raw behavioral data obtained from EthoVision XT 17 were exported and processed using custom Python scripts. Individual animal identifiers were retained throughout the preprocessing workflow to ensure traceability between behavioral measurements and biological samples.

The extracted OFT trajectories were converted into quantitative behavioral features. For each animal, zone-based features were calculated from the 3 × 3 arena configuration, including the time ratio, distance ratio, and mean velocity for each zone. In addition, global locomotor parameters were extracted, including total distance traveled, total movement time, and overall mean velocity.

To characterize spatial exploration patterns, center-zone-related features were further derived from zone 5, including center-zone occupancy and non-center exploration-related parameters. These features were combined into a unified feature matrix for subsequent computational analysis.

Before model construction, missing values were processed using mean imputation, and all behavioral features were standardized using a unified preprocessing pipeline. Feature preprocessing was implemented using Python-based tools, including Simple Imputer and Standard Scaler.

### 2.4. PCA and kNN Modeling

#### 2.4.1. Reference-State Definition and PCA Analysis

Model construction was intentionally restricted to OFT-derived features to maintain a unified multidimensional behavioral feature space and limit model complexity in the relatively small dataset. To characterize continuous behavioral variation among individual animals, standardized OFT-derived features were analyzed using PCA. PCA was applied to the unified behavioral feature matrix to reduce feature dimensionality and identify major patterns of behavioral variation.

For behavioral reference-state construction, control animals were defined as the control-like reference class, whereas untreated depression-model animals, including untreated CUMS, LPS 0.5 mg/kg, and LPS 2.0 mg/kg groups, were jointly defined as the untreated model-like reference class. This strategy was designed to capture shared OFT behavioral characteristics across different depression-like induction paradigms rather than to discriminate among etiologically distinct models.

NSC-treated animals were excluded from model training and parameter optimization. After model establishment, treated animals were projected onto the predefined behavioral space to evaluate treatment-associated shifts relative to the untreated reference states.

#### 2.4.2. kNN Classifier Construction

A k-nearest-neighbor (kNN) classifier was constructed under a strict binary setting using standardized OFT-derived features. Euclidean distance (L2 norm) was used to quantify behavioral similarity between individual animals [41].

The optimal number of neighbors (k) was determined by stratified cross-validation using balanced accuracy as the primary selection criterion. The final classifier was established with k = 3.

To minimize information leakage, data preprocessing, feature transformation, and model fitting were performed within the corresponding training procedures.

**Figure 2:**
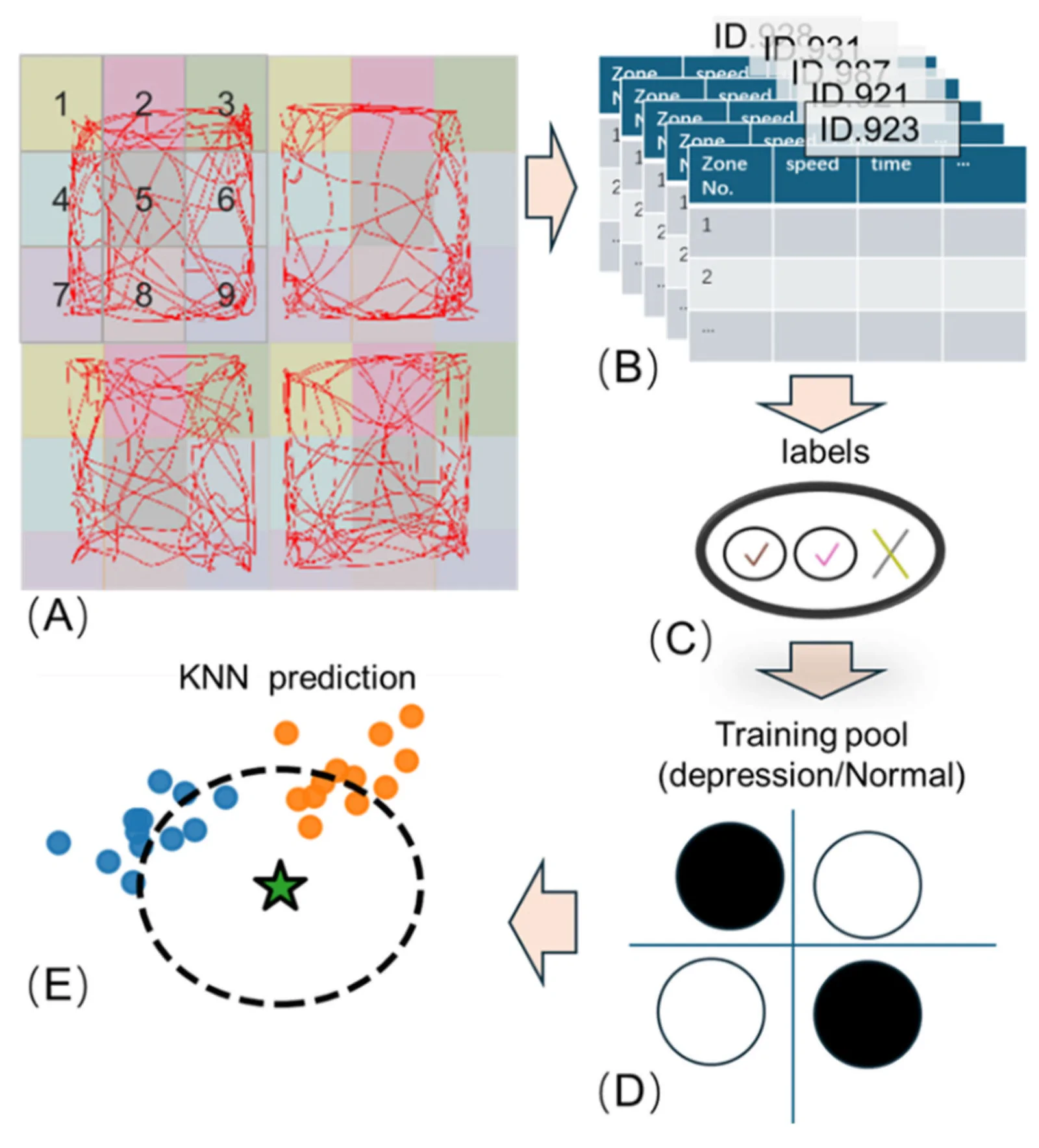
(**A**) representative OFT trajectories; (**B**) behavioral feature extraction and tabulation; (**C**) reference-state labeling; (**D**) construction of the control-like/model-like training pool; and (**E**) kNN-based behavioral inference. In panel (**E**), colored points represent the two reference classes, and the green star represents the inference sample.

The overall workflow is shown in Figure 2.

Workflow of OFT feature extraction and continuous behavioral scoring. Open-field trajectories were recorded using EthoVision XT 17 and quantified within a 3 × 3 arena grid. Zone-wise behavioral variables, together with global locomotor indices, were extracted to characterize locomotor activity and spatial exploration. After preprocessing and dimensionality reduction, control animals and untreated depression-model animals were used to define control-like and model-like behavioral states. A k-nearest-neighbor behavioral scoring classifier generated the model-derived behavioral score (p_depression), while centroid-based distance analysis yielded the relative distance metric (Δd). Together, these outputs were used to evaluate treatment-associated behavioral shifts and inter-individual variability.

#### 2.4.3. Behavioral Scoring Indices

For each inference sample, two complementary behavioral indices were calculated.

The first index, p_depression, was defined as a classifier-derived score representing the similarity of an OFT profile to the untreated model-like reference class. Higher p_depression values indicate greater similarity to the untreated model-like behavioral profile, whereas lower values indicate greater similarity to the control-like behavioral profile.

Importantly, p_depression represents an OFT-derived behavioral similarity score and should not be interpreted as a clinical probability of depression or a direct measurement of depression severity.

The second index, Δd, was calculated as the difference between the distances from each sample to the model-like and control-like reference centroids:

(1) $$\Delta d=d_{dep}-d_{ctrl}$$

where $d_{dep}$ and $d_{ctrl}$ represent the Euclidean distances between the sample and the model-like and control-like centroids, respectively. The Δd value was used to quantify the relative position of each animal within the behavioral feature space.

Together, p_depression and Δd were interpreted as quantitative indicators of treatment-associated behavioral shifts along a control-like-to-model-like OFT behavioral continuum.

### 2.5. Model Validation

To evaluate the robustness and stability of the kNN-based behavioral scoring framework, internal validation was performed using repeated stratified resampling of the reference dataset.

The reference dataset consisted exclusively of control animals and untreated depression-model animals. Intervention-associated animals were excluded from model training and validation to avoid information leakage and to ensure that the behavioral axis was established independently of treatment-related phenotypes.

In each iteration, the reference dataset was randomly divided into training and validation subsets while maintaining the original class distribution. The complete preprocessing pipeline, including missing-value imputation, feature scaling, PCA transformation, and kNN model optimization, was reconstructed independently within each iteration. Specifically, PCA transformation parameters were fitted only using the training subset and subsequently applied to the corresponding validation samples.

Model performance was evaluated on held-out validation subsets using balanced accuracy, precision, recall, F1-score, and confusion matrices. Receiver operating characteristic (ROC) curves and the area under the ROC curve (ROC-AUC) were additionally calculated as supplementary measures of classification performance.

Performance metrics were summarized across repeated iterations and reported as mean values with variability estimates. The distributions of predicted behavioral scores and classification outcomes were further examined to assess the stability of the control-like-to-model-like behavioral representation.

Given the limited biological subgroup size, this validation procedure was considered an internal robustness assessment rather than an external validation. The generalizability of the proposed behavioral phenotyping framework requires further evaluation using larger, prospectively designed cohorts, independent laboratories, different mouse strains, both sexes, and additional depression-related paradigms.

### 2.6. Biological Statistical Analyses

All biological experimental data are presented as mean ± standard deviation (SD). Statistical analyses were performed using Python 3.12-based statistical packages or equivalent statistical software.

For comparisons among experimental groups, data distribution and variance homogeneity were assessed before statistical testing. When the assumptions of normality and equal variance were satisfied, one-way analysis of variance (ANOVA) followed by Tukey’s multiple comparison test was performed. When these assumptions were not met, appropriate nonparametric tests were applied.

For comparisons between specific biological groups, Welch’s *t*-test with Holm correction and exact permutation tests were additionally performed when appropriate, considering the relatively small sample size of individual experimental groups. Effect sizes (Hedges’ g) and 95% confidence intervals were reported to provide complementary estimates of biological differences beyond *p* values.

Correlation analyses between behavioral scores, behavioral features, and biological indicators were performed using Spearman’s rank correlation analysis. For analyses involving behavioral and molecular measurements, the corresponding unit of analysis is indicated in the figure legends and text. In particular, correlations presented in Figure 10 were calculated using group-level means (*n* = 7 groups) and were therefore interpreted as exploratory associations rather than evidence of individual-level mechanistic relationships.

All statistical tests were two-sided, and *p* < 0.05 was considered statistically significant. Given the exploratory proof-of-concept design and limited subgroup size, biological findings were interpreted by considering statistical significance, effect magnitude, confidence intervals, and consistency across behavioral and molecular measurements.

## 3. Results

### 3.1. Behavioral, Neuroinflammatory, and Histopathological Alterations Across CUMS- and LPS-Induced Depression-like Models and Their Partial Attenuation by NSCs

**Figure 3:**
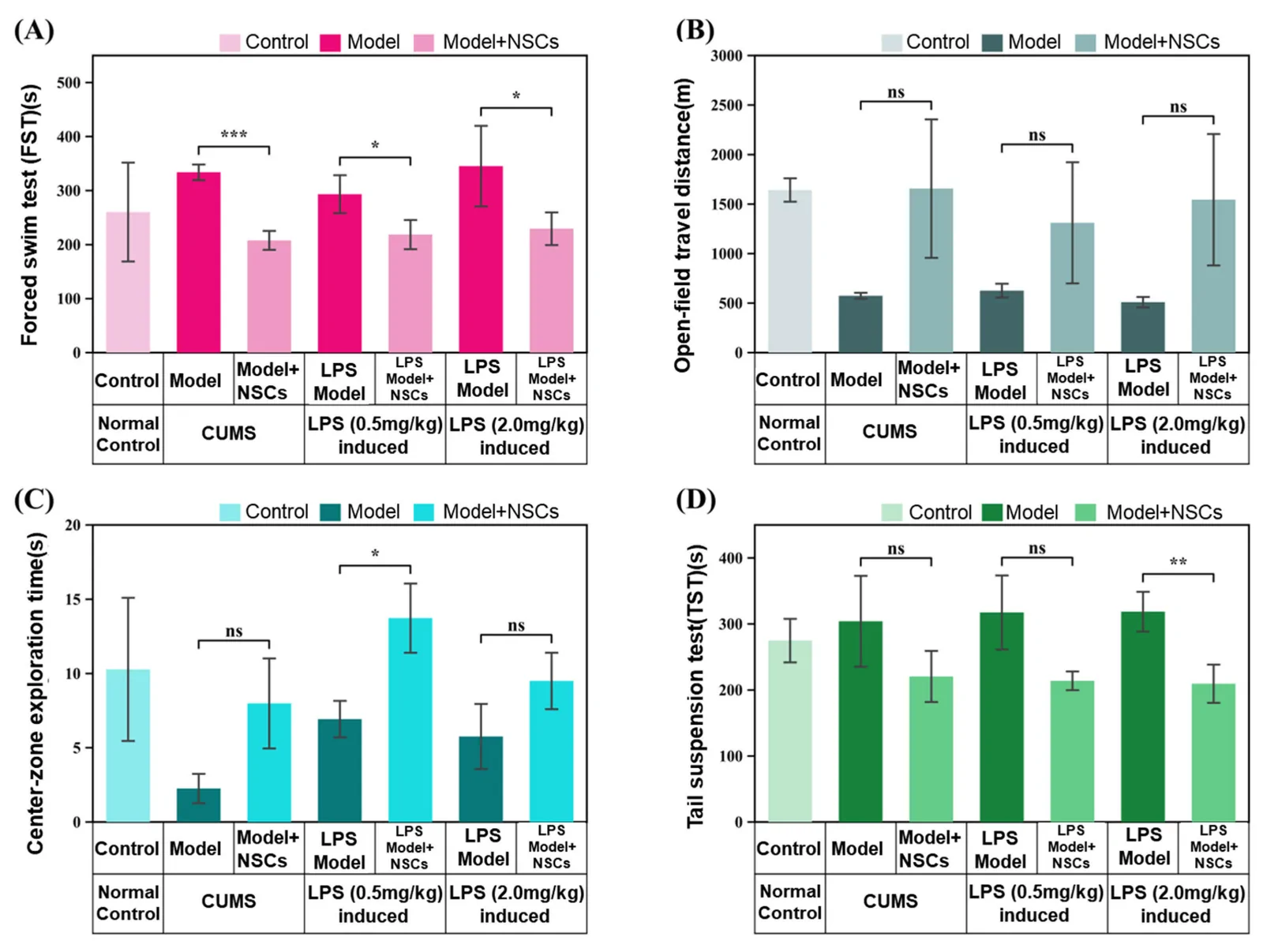
Behavioral assessments in CUMS- and LPS-induced depression-like mouse models following NSC treatment. (**A**) Immobility time in the forced swim test (FST). (**B**) Total distance traveled in the open field test (OFT), reflecting locomotor activity. (**C**) Center-zone exploration time in the OFT, reflecting exploratory and anxiety-related behavioral variation. (**D**) Immobility time in the tail suspension test (TST). Data are presented as mean ± SD (*n* = 4 animals per group). Overall group differences were evaluated using one-way ANOVA. Prespecified comparisons between each untreated model group and its corresponding NSC-treated group were evaluated using Welch’s *t*-tests with Holm correction. Brackets indicate these prespecified comparisons, and significance symbols correspond to Holm-adjusted *p* values. * *p* < 0.05, ** *p* < 0.01, *** *p* < 0.001; ns, not significant.

Significant overall group differences were detected for all four behavioral outcomes shown in Figure 3, including FST immobility time (one-way ANOVA, F(6, 21) = 4.99, *p* = 0.0026), OFT total distance (F(6, 21) = 5.96, *p* < 0.001), OFT center-zone exploration time (F(6, 21) = 7.60, *p* < 0.001), and TST immobility time (F(6, 21) = 5.61, *p* = 0.0013). Descriptively, untreated CUMS- and LPS-induced groups generally showed greater immobility in the FST and TST and reduced locomotor and exploratory activity in the OFT compared with the control group.

Prespecified comparisons between each untreated model group and its corresponding NSC-treated group showed outcome-dependent treatment-associated differences. In the FST, immobility time was significantly lower in CUMS+NSCs than in CUMS (Holm-adjusted *p* < 0.001), in LPS0.5+NSCs than in LPS0.5 (adjusted *p* = 0.033), and in LPS2.0+NSCs than in LPS2.0 (adjusted *p* = 0.046). For OFT total distance, none of the three prespecified comparisons remained significant after Holm correction (all adjusted *p* = 0.156). For OFT center-zone exploration time, a significant increase was detected for LPS0.5+NSCs versus LPS0.5 (adjusted *p* = 0.014), whereas the CUMS and LPS2.0 comparisons did not reach statistical significance (both adjusted *p* = 0.054). In the TST, a significant reduction in immobility time was detected for LPS2.0+NSCs versus LPS2.0 (adjusted *p* = 0.0059), whereas the CUMS and LPS0.5 comparisons were not statistically significant (adjusted *p* = 0.091 and 0.061, respectively). Overall, these findings indicate treatment-associated behavioral shifts whose magnitude and statistical support varied across behavioral assays and induction paradigms.

**Figure 4:**
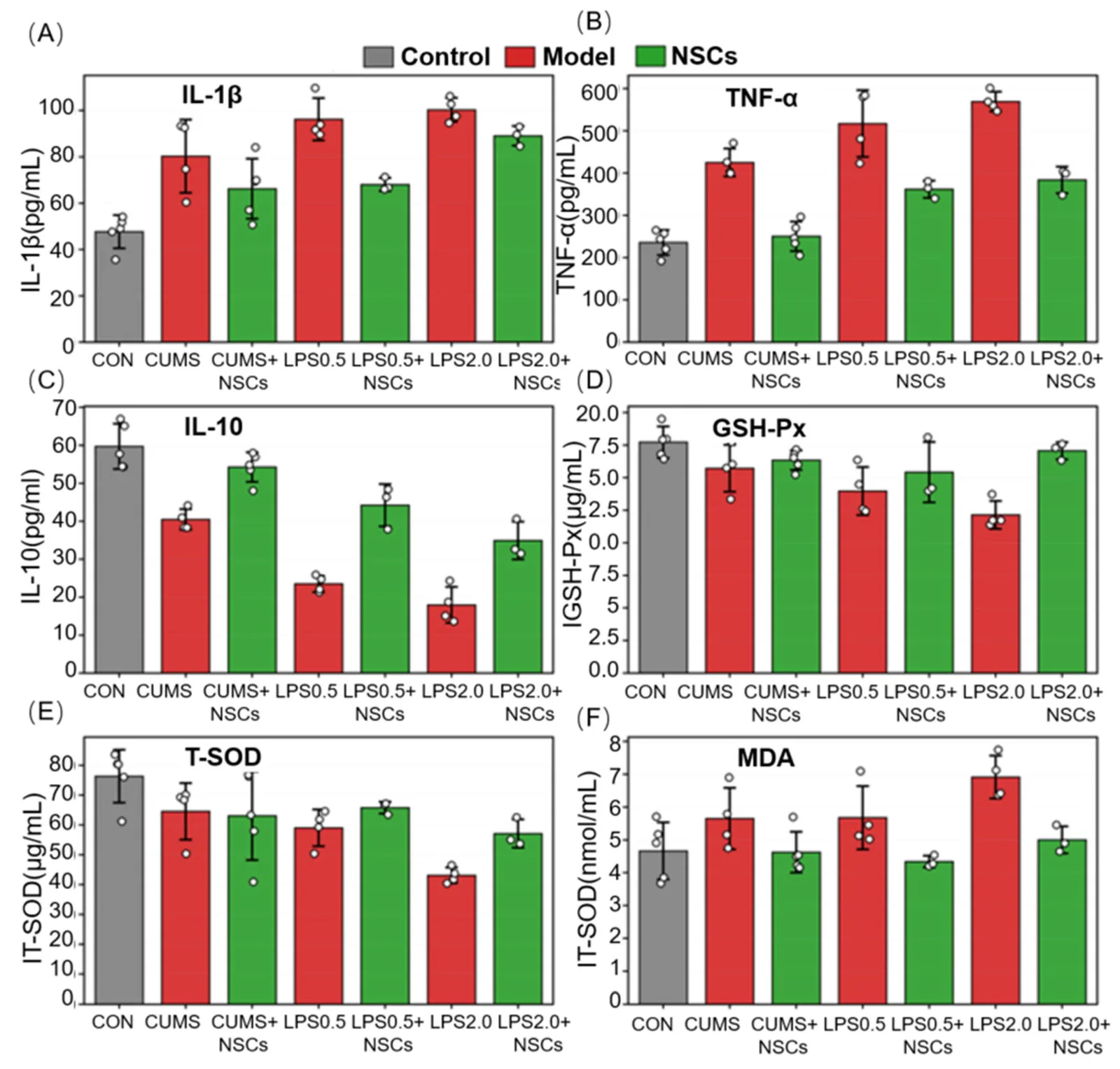
Group-wise changes in neuroinflammatory and oxidative-stress-related biochemical markers following NSC treatment in CUMS- and LPS-induced mouse models. (**A**–**F**) Hippocampal levels of (**A**) IL-1β, (**B**) TNF-α, (**C**) IL-10, (**D**) GSH-Px, (**E**) T-SOD, and (**F**) MDA. Individual circles represent biological samples, and bars and error bars represent mean ± SD. Biological sample sizes were CON, *n* = 5; CUMS, *n* = 4; CUMS+NSCs, *n* = 4; LPS0.5, *n* = 4; LPS0.5+NSCs, *n* = 2; LPS2.0, *n* = 4; and LPS2.0+NSCs, *n* = 3. Overall group differences were evaluated using Welch’s ANOVA because the available biological sample sizes differed among groups. Prespecified comparisons between each untreated model group and its corresponding NSC-treated group were additionally evaluated using Welch’s *t*-tests with Holm correction.

In addition to behavioral evaluation, inflammatory cytokines and oxidative stress-related markers were measured as biological indicators of model establishment. IL-1β and TNF-α were selected as representative pro-inflammatory mediators, whereas IL-10 reflects anti-inflammatory regulation. GSH-Px and T-SOD represent antioxidant defense capacity, while MDA reflects lipid peroxidation and oxidative damage. Neuroinflammatory and oxidative-stress-related measures showed parallel group-wise changes (Figure 4). Compared with controls, untreated model groups exhibited higher levels of pro-inflammatory cytokines, including IL-1β and TNF-α, together with lower IL-10. Model groups also showed reduced antioxidant-related indices and increased lipid peroxidation, consistent with altered inflammatory and oxidative balance. NSC-treated groups tended to show partial normalization of these measures relative to their corresponding untreated groups.

**Figure 5:**
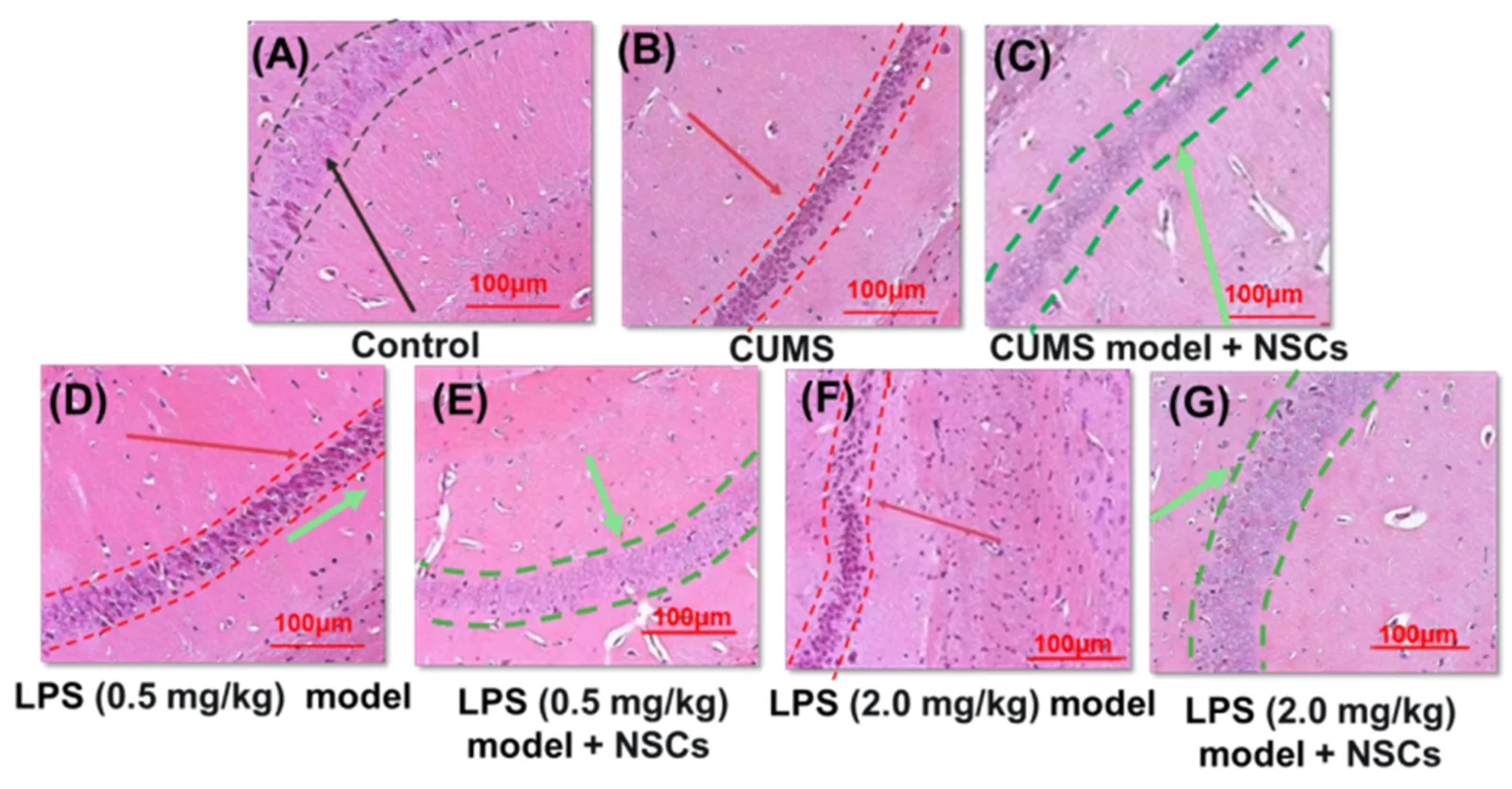
Representative hematoxylin and eosin (H&E)-stained hippocampal sections from control, CUMS, and LPS-induced mice with or without NSC treatment. (**A**) Control; (**B**) CUMS; (**C**) CUMS + NSCs; (**D**) LPS 0.5 mg/kg; (**E**) LPS 0.5 mg/kg + NSCs; (**F**) LPS 2.0 mg/kg; and (**G**) LPS 2.0 mg/kg + NSCs. Images are presented for qualitative morphological comparison only. No formal quantitative histopathological scoring was performed. Black, red, and green arrows and dashed outlines indicate representative morphological features highlighted for visual comparison only; these annotations were not used for quantitative histopathological scoring. Scale bar = 100 μm.

Qualitative examination of representative H&E-stained hippocampal sections suggested morphological differences among the experimental groups (Figure 5). Because no formal blinded morphometric quantification was performed, these observations are presented as descriptive supportive findings only and should not be interpreted as quantitative evidence of histological restoration.

Neuroinflammatory and oxidative-stress-related measures showed significant overall differences among the experimental groups (Figure 4). Because the numbers of available biological samples differed among groups, Welch’s ANOVA was used for these analyses. Significant overall group effects were detected for IL-1β (F(6, 7.94) = 30.38, *p* < 0.001), IL-10 (F(6, 6.48) = 37.92, *p* < 0.001), TNF-α (F(6, 6.95) = 51.52, *p* < 0.001), GSH-Px (F(6, 6.62) = 8.34, *p* = 0.0078), T-SOD (F(6, 7.93) = 25.10, *p* < 0.001), and MDA (F(6, 8.04) = 6.55, *p* = 0.0091).

Prespecified comparisons between each untreated model group and its corresponding NSC-treated group were further evaluated using Welch’s *t*-tests with Holm correction. For IL-1β, a significant reduction was detected in the LPS0.5+NSCs group compared with the LPS0.5 group (Holm-adjusted *p* = 0.0171), whereas the CUMS and LPS2.0 comparisons did not reach the adjusted significance threshold (*p* = 0.1679 and *p* = 0.0514, respectively). IL-10 was significantly increased in CUMS+NSCs versus CUMS (adjusted *p* = 0.0095) and in LPS2.0+NSCs versus LPS2.0 (adjusted *p* = 0.0172), whereas the LPS0.5 comparison was not significant (*p* = 0.1398).

For oxidative-stress-related indices, MDA was significantly reduced in LPS2.0+NSCs compared with LPS2.0 (adjusted *p* = 0.0160), and GSH-Px was significantly increased in the same comparison (adjusted *p* = 0.0022). No Holm-adjusted comparison for T-SOD reached statistical significance, although the LPS2.0 versus LPS2.0+NSCs comparison approached the conventional threshold (adjusted *p* = 0.0608). TNF-α showed significant reductions following NSC treatment in all three model conditions (CUMS+NSCs vs. CUMS, adjusted *p* = 0.0011; LPS0.5+NSCs vs. LPS0.5, adjusted *p* = 0.0216; LPS2.0+NSCs vs. LPS2.0, adjusted *p* = 0.0032).

Exact permutation tests were additionally performed as small-sample sensitivity analyses. These supported the CUMS-associated changes in IL-10 and TNF-α (both permutation *p* = 0.0286), whereas the permutation tests for the LPS-based treatment comparisons did not reach *p* < 0.05. Given the limited and unequal numbers of biological samples available for the biochemical assays, these marker-specific treatment comparisons were interpreted cautiously and primarily as complementary biological evidence rather than definitive mechanistic evidence.

### 3.2. OFT Behavioral Features Distribute Along a Continuous Spectrum in PCA Space

**Figure 6:**
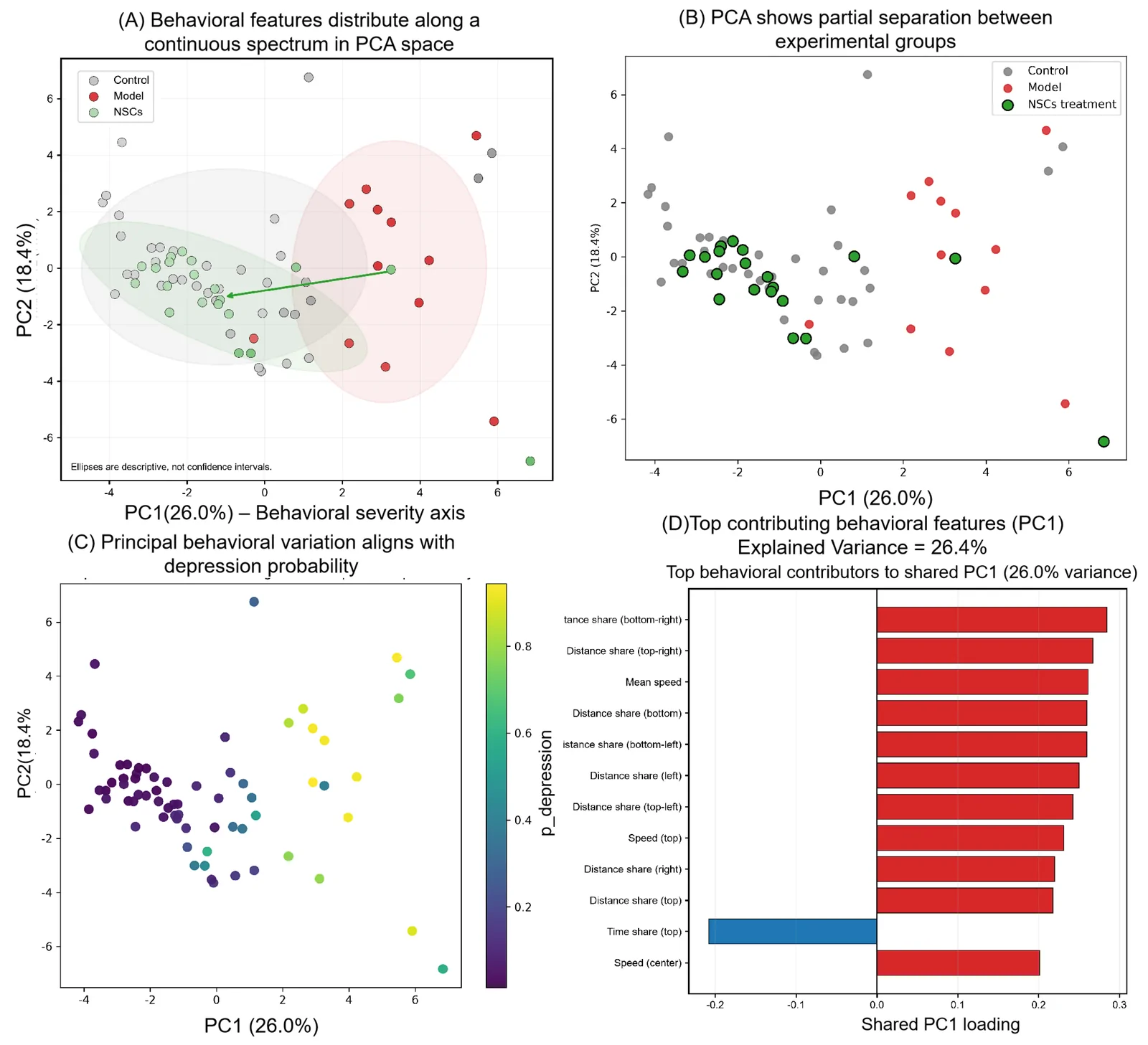
OFT-derived behavioral features define a continuous spectrum of depression-like states in PCA space. (**A**) PCA projection of OFT-derived behavioral features shows that samples are distributed along a continuous spectrum rather than forming sharply separated categories. Control, untreated model, and NSC-treated animals occupy partially overlapping regions, and the arrow indicates the overall shift of NSC-treated animals toward a more control-like behavioral state. The horizontal arrow denotes the behavioral severity axis along PC1. the green arrow indicates the overall direction of the NSC-associated shift toward a more control-like behavioral state. (**B**) PCA plot colored by experimental group illustrates partial separation between control, untreated model, and NSC-treated animals, with substantial overlap of NSC-treated animals with both ends of the behavioral spectrum. (**C**) PCA plot colored by the model-derived behavioral score (p_depression) reveals a smooth gradient along the principal behavioral axis, supporting the interpretation of PC1 as a continuous coordinate of behavioral severity. (**D**) PC1 loading plot identifies the OFT-derived features that contributed most strongly to the principal behavioral axis. Positive and negative loadings indicate features associated with opposite directions along the behavioral severity continuum. PC1 and PC2 explained 26.4% and 26.0% of the total variance, respectively.

To examine the global organization of OFT-derived behavioral variation, we performed PCA on the multidimensional behavioral feature set. As shown in Figure 6A, samples were distributed along a continuous trajectory in PCA space rather than forming sharply separated clusters, suggesting that depression-like OFT phenotypes in the present dataset are better represented as a continuum than as strictly discrete categories. Along this trajectory, control animals were located predominantly at one end, whereas untreated model animals were shifted toward the opposite end. In behavioral terms, movement along this axis corresponded to a transition from a more preserved exploratory profile toward a more model-like pattern characterized by reduced locomotor activity and altered spatial exploration. NSC-treated animals occupied intermediate regions between these two extremes, consistent with partial behavioral recovery rather than abrupt normalization.

This overall organization was further supported by the group-colored PCA projection (Figure 6B), which showed only partial separation among the control, untreated model, and NSC-treated animals. In particular, NSC-treated animals overlapped with both ends of the behavioral spectrum, indicating intermediate phenotypes and substantial inter-individual variability rather than sharp category transitions.

When samples were colored according to the model-derived behavioral score (p_depression), a smooth gradient emerged along PC1 (Figure 6C), further supporting the interpretation of this axis as a continuous coordinate of behavioral severity. Thus, the principal behavioral variation captured in PCA space was aligned with the probabilistic state estimate generated by the scoring framework.

The PC1 loading profile identified multiple OFT-derived features contributing to this principal axis (Figure 6D), indicating that the observed continuum was shaped by coordinated variation in locomotor activity and spatial exploration rather than by any single isolated parameter. Together, these findings indicate that OFT-derived behavioral features in the present dataset are organized along a continuous behavioral spectrum, thereby providing the basis for subsequent reference-state scoring of NSC-associated behavioral shifts.

### 3.3. A Quantitative Behavioral Axis Captures Graded Model-like States and NSC-Associated Shifts

**Figure 7:**
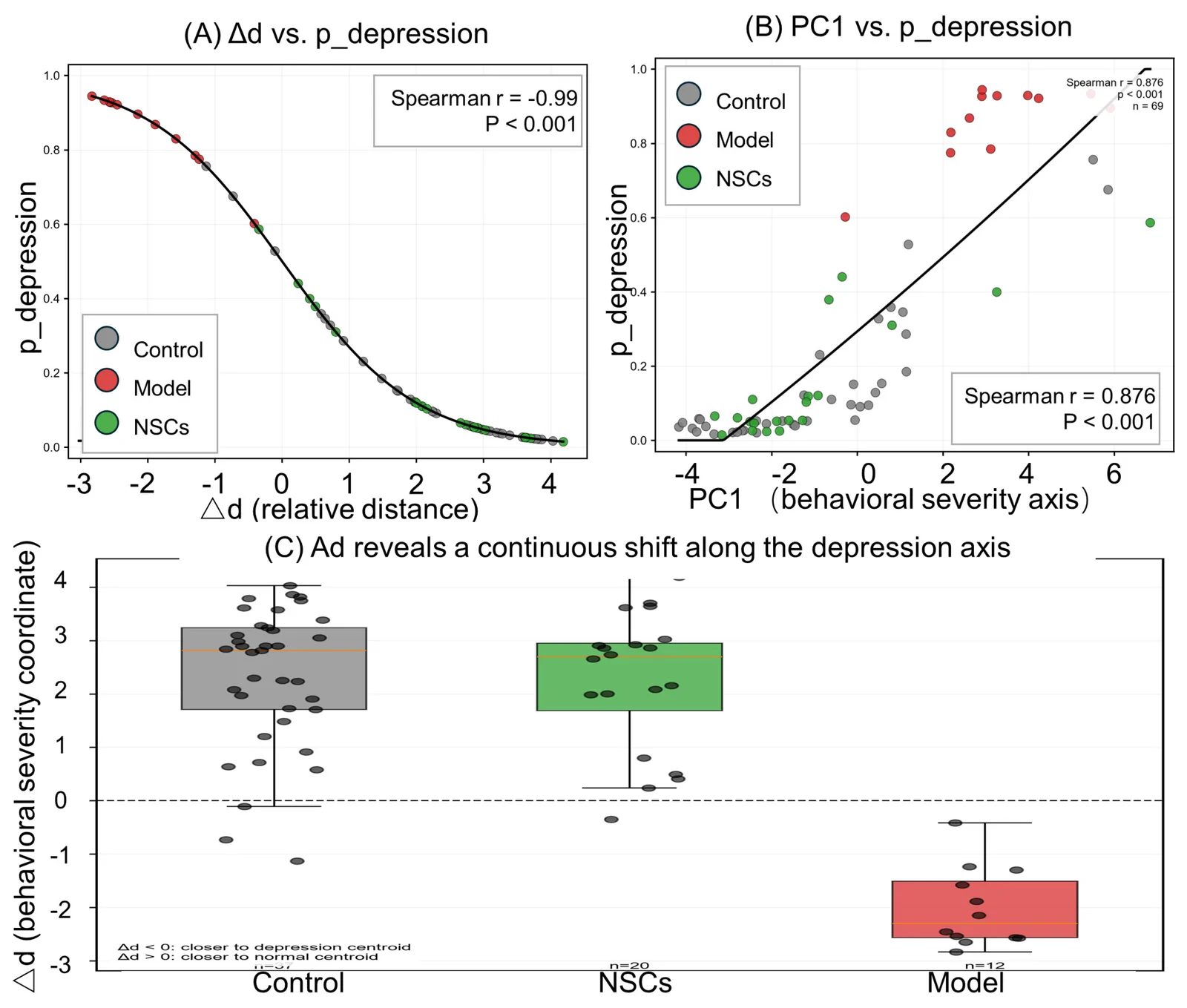
Quantitative behavioral indices capture graded depression-like states and NSC-associated shifts. (**A**) Relationship between the centroid-based behavioral coordinate (Δd) and the model-derived behavioral score (p_depression). The strong inverse association indicates that these two indices provide concordant quantitative descriptions of position along the behavioral continuum. (**B**) Relationship between PC1 and p_depression. The strong positive association indicates that the principal behavioral axis identified by PCA is aligned with the probabilistic estimate of model-like state derived from kNN inference. (**C**) Group-wise distribution of Δd across control, untreated model, and NSC-treated animals. Untreated model animals occupy the model-like end of the behavioral axis, whereas NSC-treated animals are positioned between untreated model and control animals, indicating partial behavioral recovery. Data points represent individual animals. Correlation coefficients were calculated using Spearman correlation analysis.

To translate the continuous behavioral organization observed in PCA space into interpretable quantitative indices, we next examined the relationships among Δd, p_depression, and PC1. As shown in Figure 7A, Δd was strongly inversely correlated with p_depression, indicating that these two measures captured the same underlying behavioral continuum from opposite directions. In practical terms, animals with lower or more negative Δd values tended to have higher p_depression, corresponding to OFT profiles more similar to the untreated model-like state. Conversely, animals with larger positive Δd values tended to have lower p_depression, indicating a more control-like exploratory profile.

A similarly strong relationship was observed between PC1 and p_depression (Figure 7B), supporting the interpretation that the principal behavioral variation identified by PCA is aligned with the model-derived estimate of model-like state. Thus, the model-like behavioral axis defined in PCA space was not an abstract statistical construct, but corresponded closely to the probability that a given animal’s OFT phenotype resembled the untreated model-like reference.

Group-wise analysis of Δd further illustrated this graded organization (Figure 7C). Control animals were distributed toward the control-like end of the behavioral axis, whereas untreated model animals occupied the opposite end, indicating stronger model-like behavioral features. NSC-treated animals were positioned between these two groups, consistent with treatment-associated shift toward the control-like state rather than abrupt normalization. Importantly, the spread of individual data points within the NSC-treated group suggested substantial inter-individual variability in treatment-associated behavioral shift.

Together, these findings indicate that the proposed scoring framework converts multidimensional OFT behavior into a quantitative behavioral axis that captures graded model-like states and treatment-associated transitions. In this framework, higher p_depression and lower Δd indicate greater similarity to the untreated model-like state, whereas lower p_depression and higher Δd indicate a shift toward a more control-like behavioral phenotype.

### 3.4. Quantitative Behavioral Scoring Reveals NSC-Associated Shifts and Inter-Individual Variability Across Depression-like Conditions

**Figure 8:**
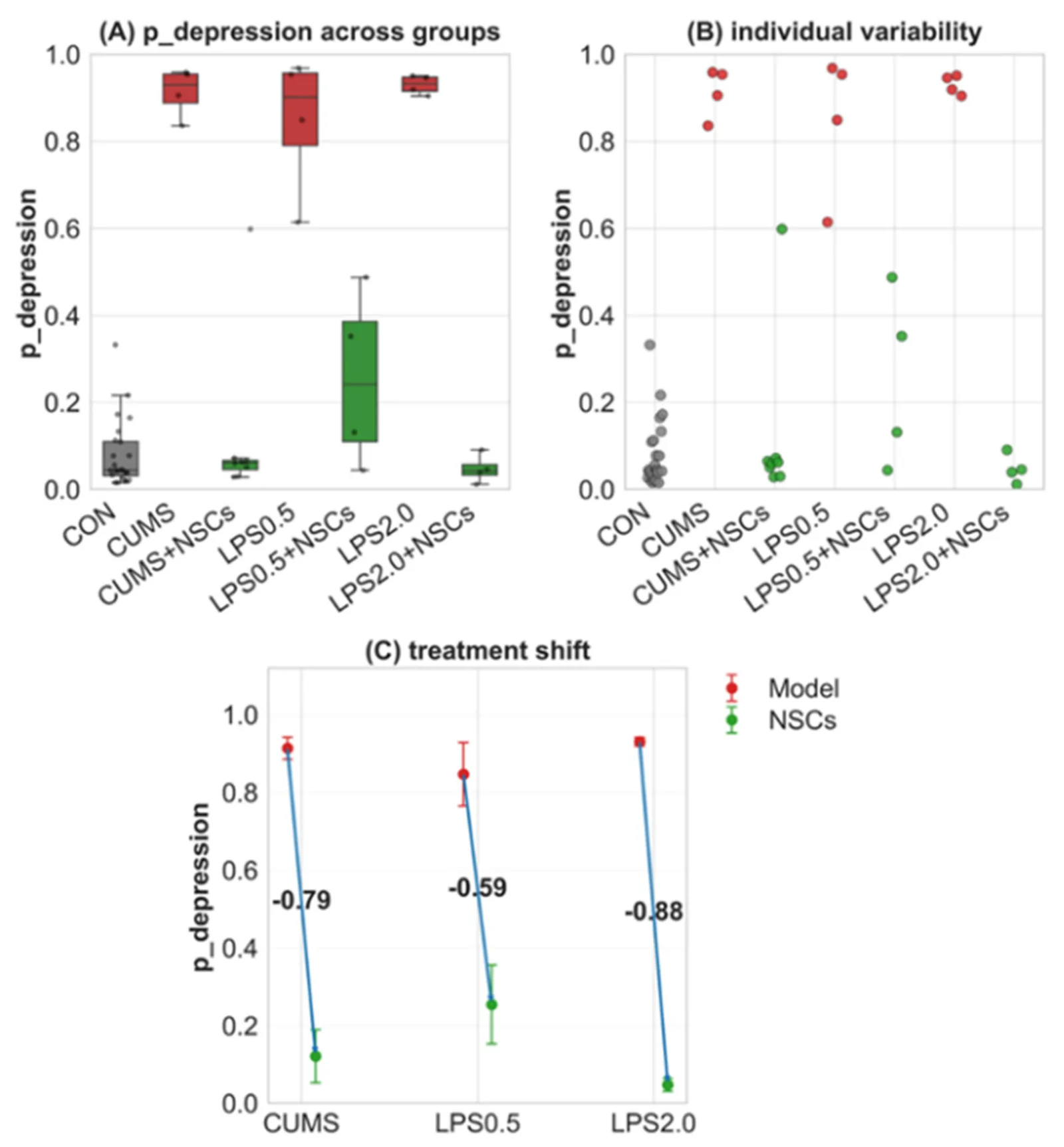
Quantitative behavioral scoring reveals treatment-associated shifts and inter-individual variability. (**A**) Distribution of the model-derived behavioral score (p_depression) across experimental groups. Untreated CUMS- and LPS-induced groups show consistently high p_depression values, indicating OFT profiles more similar to the untreated model-like state, whereas control animals show low values. The corresponding NSC-treated groups display reduced p_depression, indicating a shift toward a more control-like exploratory profile. (**B**) Individual-level variability in p_depression across groups. Each point represents a single animal, highlighting substantial heterogeneity in the extent of treatment-associated behavioral shift. (**C**) Quantification of treatment-associated changes in p_depression. Mean values (±SD) are shown for untreated model groups and their corresponding NSC-treated groups, with arrows indicating the direction and magnitude of behavioral shift following treatment. Colors: gray = control, red = untreated model, green = NSC-treated.

To further evaluate treatment-associated behavioral change at the individual level, we applied the model-derived behavioral score (p_depression) across all experimental groups. As shown in Figure 8A, untreated CUMS- and LPS-induced groups consistently exhibited elevated p_depression values compared with controls, indicating that their OFT phenotypes were positioned closer to the untreated model-like state. In contrast, the corresponding NSC-treated groups showed lower p_depression values, suggesting that treatment shifted the overall behavioral pattern away from the model-like low-activity, low-exploration profile and toward a more control-like state.

At the individual level, substantial variability was observed within treatment groups (Figure 8B). Although most NSC-treated animals showed lower p_depression values than their corresponding untreated model groups, the extent of this shift differed markedly among individuals. This pattern indicates that the proposed behavioral scoring framework not only captures group-level treatment-associated change but also preserves heterogeneity in treatment response that may be obscured by categorical group comparisons alone.

To quantify treatment-associated behavioral shift more directly, we compared mean p_depression values between untreated model groups and their corresponding NSC-treated groups (Figure 8C). Downward shifts in p_depression were observed across all three model conditions, indicating that NSC treatment was associated with movement away from the untreated model-like state. The largest shift was observed in the LPS 2.0 mg/kg condition, whereas the shift was more moderate in the LPS 0.5 mg/kg condition. CUMS-treated animals also showed a clear downward shift, further supporting that the same scoring framework can capture treatment-associated behavioral change in a distinct stress-based induction paradigm.

Together, these findings indicate that quantitative OFT-based scoring provides an interpretable measure of whether individual animals remain model-like or shift toward a more control-like behavioral state after treatment. In this framework, p_depression serves not only as a group-discriminating metric but also as a sensitive readout of treatment-associated behavioral shift and inter-individual variability.

### 3.5. Quantitative Behavioral Scoring Reveals Treatment-Associated Shifts and Is Supported by Expanded-Cohort Robustness Analysis

**Figure 9:**
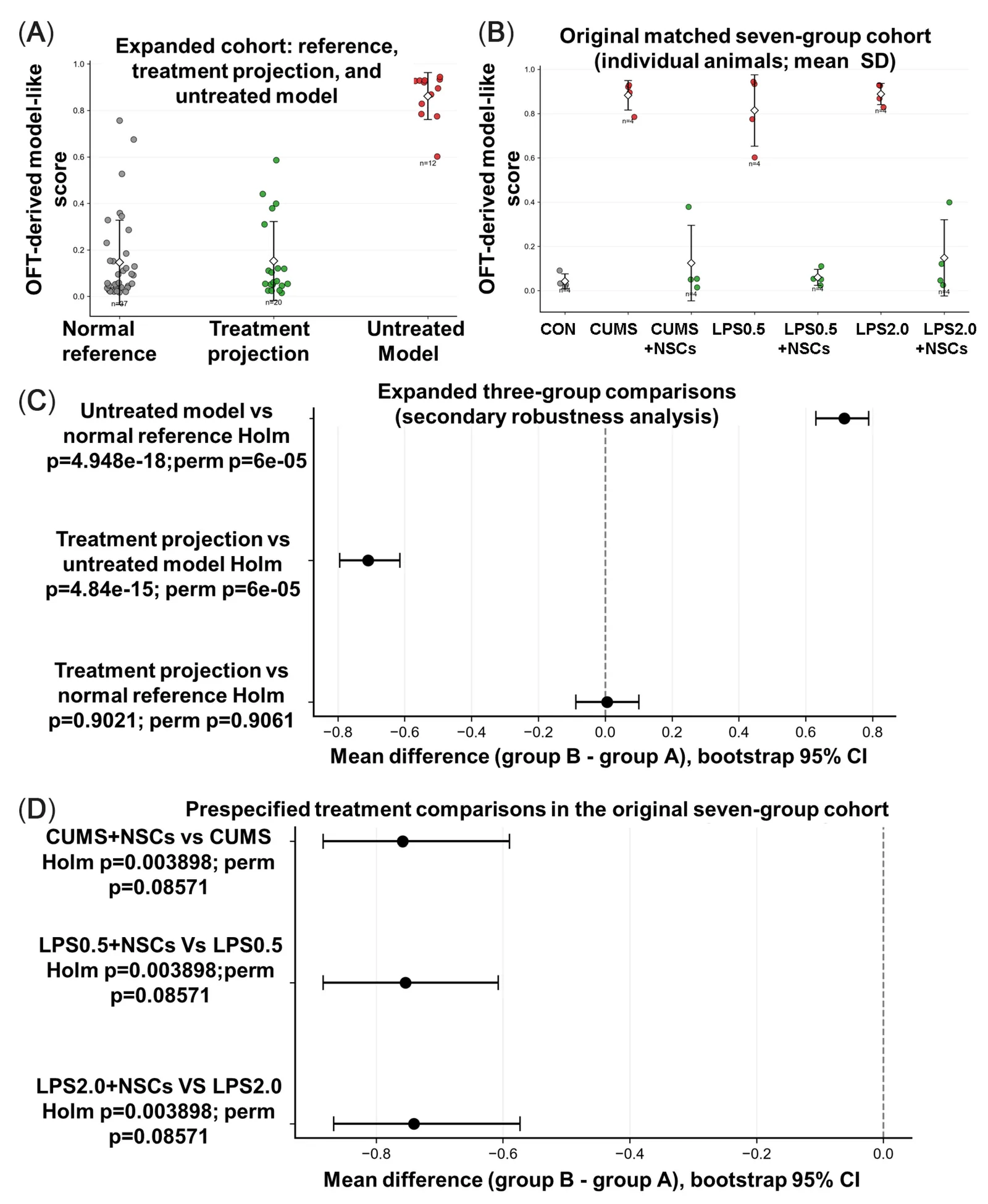
OFT-derived model-like scores and treatment-associated shifts in the original and expanded cohorts. (**A**) Individual OFT-derived model-like scores in the expanded cohort. Points represent individual animals; diamonds and error bars indicate group means ± SD. (**B**) Individual scores in the original seven-group cohort. (**C**) Pairwise comparisons among the expanded normal-reference, untreated-model, and intervention-associated populations. (**D**) Prespecified comparisons between each untreated model group and its corresponding NSC-treated group. Points in forest plots represent mean differences (group B − group A), and horizontal bars represent bootstrap 95% confidence intervals. Holm-adjusted *p* values were obtained from Welch’s *t*-tests, and exact permutation *p* values are additionally reported as small-sample sensitivity analyses.

To further quantify treatment-associated behavioral changes, we examined the OFT-derived model-like score at both the original seven-group level and in an expanded behavioral cohort. In the original matched seven-group cohort, untreated CUMS-, LPS0.5-, and LPS2.0-induced animals consistently exhibited high model-like scores, whereas the corresponding NSC-treated groups showed marked downward shifts toward the control-like range (Figure 9A).

Prespecified comparisons between each untreated model group and its corresponding NSC-treated group showed large and directionally consistent reductions in the model-like score (Figure 9B). The Holm-adjusted parametric comparisons were significant for CUMS+NSCs versus CUMS, LPS0.5+NSCs versus LPS0.5, and LPS2.0+NSCs versus LPS2.0 (all Holm-adjusted *p* = 0.003898). Bootstrap 95% confidence intervals for the mean differences did not cross zero, indicating large treatment-associated shifts in all three induction paradigms. However, the corresponding exact permutation tests yielded *p* = 0.08571 for each comparison. Given the small subgroup size (*n* = 4), these model-specific results were therefore interpreted as exploratory evidence of large and directionally consistent treatment-associated behavioral shifts rather than as definitive confirmatory evidence of efficacy.

To assess whether this pattern remained evident in a larger dataset, a secondary robustness analysis was performed using all available OFT behavioral data. The expanded cohort comprised 37 normal-reference animals, 12 untreated model animals, and 20 intervention-associated animals (Figure 9C). Untreated model animals showed substantially higher model-like scores than the normal-reference population (Holm-adjusted *p* = 4.948 × 10^−18^; permutation *p* = 6.0 × 10^−5^). Intervention-associated animals also showed markedly lower scores than untreated model animals (Holm-adjusted *p* = 4.84 × 10^−15^; permutation *p* = 6.0 × 10^−5^) (Figure 9D). In contrast, no evidence of a difference was detected between the intervention-associated and normal-reference populations (Holm-adjusted *p* = 0.9021; permutation *p* = 0.9061).

Together, these analyses indicate that the treatment-associated displacement observed in the original seven-group cohort was also evident at the expanded-cohort level. Importantly, the absence of a detectable difference between intervention-associated and normal-reference animals should not be interpreted as statistical equivalence or complete behavioral normalization, but rather as indicating that the present analysis did not detect a group-level difference between these populations.

### 3.6. Exploratory Group-Level Correspondence Between Behavioral Scores and Neuroinflammatory Profiles

**Figure 10:**
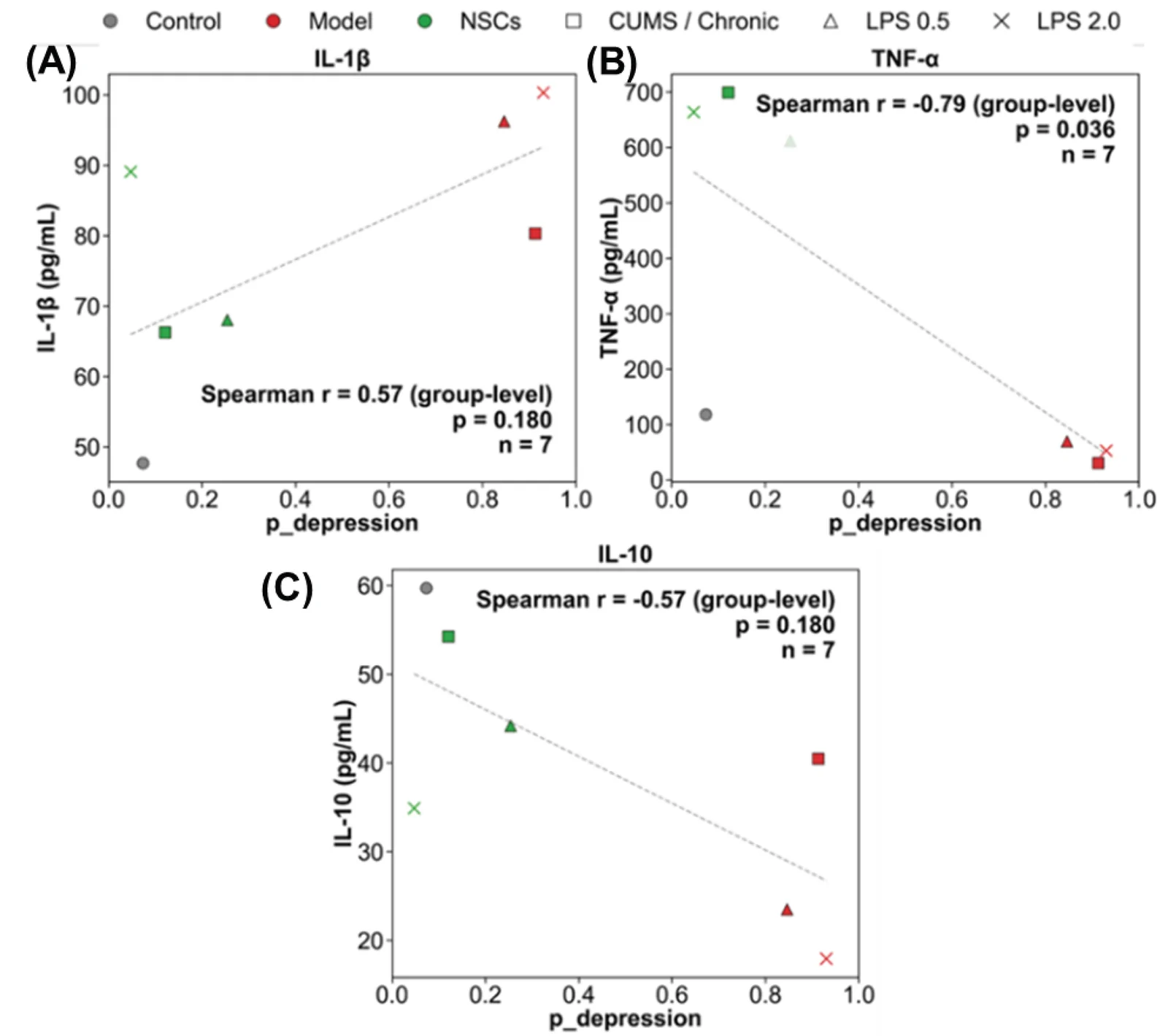
Exploratory group-level correspondence between behavioral severity and neuroinflammatory profiles. (**A**) Association between IL-1β and the model-derived behavioral score (p_depression). (**B**) Association between TNF-α and p_depression. (**C**) Association between IL-10 and p_depression. Each point represents the mean value of one experimental group (*n* = 7 groups). Colors indicate experimental conditions (gray, control; red, untreated model; green, NSC-treated), and marker shapes denote induction paradigms (square, CUMS/chronic; triangle, LPS 0.5 mg/kg; cross, LPS 2.0 mg/kg). Spearman correlation analysis was performed at the group level. These analyses are intended to illustrate exploratory group-level correspondence and should not be interpreted as evidence of individual-level coupling or causal biological linkage.

To examine the potential biological relevance of the proposed behavioral scoring framework, we assessed the relationships between the model-derived behavioral score (p_depression) and neuroinflammatory markers at the group level (Figure 10). Because these analyses were performed on group means rather than individual paired measurements, they should be interpreted as exploratory correspondence analyses.

IL-1β showed a moderate positive association with p_depression (Figure 10A), indicating that groups with more model-like OFT phenotypes tended to show higher pro-inflammatory signaling. However, this relationship did not reach statistical significance in the present dataset and should therefore be interpreted cautiously. IL-10 showed a negative trend with p_depression (Figure 10C), suggesting that groups with more model-like behavioral profiles tended to show lower anti-inflammatory signaling, although this trend was likewise not statistically significant.

TNF-α showed a significant inverse association with p_depression across groups (Figure 10B). Given the limited number of group-level observations and the heterogeneity across induction paradigms, this finding should not be overinterpreted as a simple linear relationship between behavioral severity and a single inflammatory mediator. Rather, it may reflect paradigm-dependent inflammatory dynamics or temporal differences between chronic stress- and LPS-related biological responses.

Despite these limitations, the overall pattern suggests that the behavioral shift captured by the proposed scoring framework is not isolated from biological context, but may occur in parallel with broader inflammatory-state differences across experimental conditions. These findings provide preliminary support for the biological relevance of the proposed quantitative behavioral framework while highlighting the need for validation in larger cohorts and with individual-level paired behavioral and molecular measurements.

## 4. Discussion

In the present study, we developed a continuous OFT-based behavioral phenotyping framework to evaluate depression-like states and NSC-associated behavioral shifts in mice. Consistent with recent CUMS and LPS studies, untreated model animals showed altered locomotor and exploratory behavior [42,43,44]. Although CUMS and LPS involve distinct induction mechanisms, their OFT profiles partially overlapped within a model-like behavioral space. Importantly, CUMS and LPS were not considered biologically identical models in this study. CUMS was used to represent a chronic stress-related paradigm, whereas LPS was used as an inflammation-associated acute challenge model. The rationale for including both paradigms was to examine whether etiologically distinct models could converge on partially shared behavioral characteristics that may be captured by a common OFT-derived behavioral representation. NSC-treated animals shifted toward a more control-like profile, suggesting that treatment-associated behavioral improvement may occur along a graded continuum rather than as an all-or-none response.

The accompanying inflammatory, oxidative-stress-related, and hippocampal changes are also consistent with evidence linking chronic stress and LPS exposure to neuroinflammation, oxidative imbalance, and neural alterations associated with depression-like behaviors [43,44]. In the present study, these biological measurements were not incorporated into the machine-learning model but were used as complementary evidence to support successful model establishment and to examine whether behavioral changes identified by the OFT-based framework were directionally consistent with underlying biological alterations. NSC-related interventions, including NSC-derived extracellular vesicles, have shown immunomodulatory and neuroprotective effects [45]. Therefore, NSCs were selected as an intervention strategy because of their potential effects on inflammatory regulation, trophic support, and neural repair beyond conventional monoaminergic antidepressant mechanisms. Nevertheless, the parallel behavioral and biological changes observed here indicate association rather than a demonstrated causal mechanism.

Recent multivariate and AI-based studies further demonstrate the value of multidimensional behavioral analysis for resolving inter-individual heterogeneity and model-specific behavioral patterns [5,22]. Accordingly, the novelty of the present study lies not in OFT, PCA, or kNN individually, but in integrating them into an interpretable control-like-to-model-like continuum that quantifies treatment-associated shifts at the individual-animal level. Unlike conventional categorical comparisons, this framework preserves individual behavioral variation and allows partially recovered or intermediate phenotypes to be represented quantitatively. This framework should therefore be regarded as an exploratory quantitative phenotyping approach rather than a validated measure of depression severity.

Several limitations should be acknowledged. First, the biological subgroup size was small (*n* = 4/group), and no formal a priori power calculation had been performed for the original exploratory experiment. Consequently, these subgroup-level biological comparisons have limited statistical precision and should be regarded as exploratory rather than confirmatory. Larger prospectively powered cohorts will be required to validate the observed effects. Although repeated cross-validation, bootstrap analysis, and permutation-based feature assessment were performed to evaluate model stability, these internal validation approaches cannot replace independent external validation. Therefore, potential overfitting and limited generalizability remain important considerations.

Second, only male mice were included, and sex was not examined as an experimental factor. Given established sex-dependent differences in stress susceptibility, inflammatory responses, and behavioral responses to CUMS and LPS [42,46,47], the present findings may not generalize to females. Future studies should therefore validate the OFT-derived behavioral continuum and NSC-associated behavioral shifts in both sexes.

Third, combining CUMS and LPS into a shared reference may obscure model-specific behavioral features. The present framework was designed to capture shared behavioral states rather than to imply identical pathological mechanisms between chronic stress and inflammation-associated models. Finally, OFT measures are not specific to depression-related processes and may also reflect anxiety-like behavior, sickness responses, or general activity changes. Similarly, FST and TST were used as complementary behavioral assessments to support model characterization rather than as direct components of the continuous OFT-based prediction framework. Validation in larger and independent cohorts will be required to establish the generalizability and reproducibility of this approach.

## 5. Conclusions

In this study, we developed a quantitative OFT-based behavioral scoring framework for representing model-like OFT phenotypes along a continuous behavioral spectrum rather than as discrete categories. By integrating open-field-test-derived features with principal component analysis and k-nearest-neighbor inference, we derived two related indices, Δd and p_depression, that positioned individual animals along a control-like-to-model-like behavioral continuum.

Using control animals and untreated CUMS- and LPS-induced mice as reference states, this framework enabled evaluation of whether NSC-treated animals remained closer to a model-like state or shifted toward a more control-like exploratory profile. In behavioral terms, higher p_depression and lower Δd corresponded to OFT phenotypes that more closely resembled untreated model-like mice, whereas lower p_depression and higher Δd indicated more control-like exploratory behavior. NSC-treated animals showed measurable shifts away from the model-like state while preserving substantial inter-individual variability that may be difficult to resolve using conventional categorical comparisons alone.

Because the model-like reference class was defined jointly by untreated CUMS- and LPS-induced mice, the present framework captured shared behavioral features across distinct induction paradigms while focusing treatment evaluation specifically on the NSC-treated groups. Group-level associations between behavioral scores and neuroinflammatory markers further suggested preliminary biological relevance, although these findings require cautious interpretation and validation in larger datasets with individual-level paired measurements.

Overall, the present results support continuous behavioral scoring as a useful proof-of-concept approach for quantitative phenotyping and treatment-response assessment in preclinical depression research. Validation in larger and independent cohorts will be important to determine the generalizability of this framework across paradigms and experimental settings.

## Figures and Tables

**Table 1:** Composition of the behavioral analysis populations used for continuous OFT-based phenotyping.

Population	*n*	Composition
Normal-reference	37	24 animals from the primary cohort + 13 additional untreated animals
Untreated model	12	CUMS, LPS0.5, LPS2.0 (*n* = 4 each)
Intervention-associated	20	NSC- and melatonin-treated animals under different model conditions

## Data Availability

The data presented in this study are available on request from the corresponding author due to ethical and institutional restrictions.
